# Increased Endoparasite Infection in Late-Arriving Individuals of a Trans-Saharan Passerine Migrant Bird

**DOI:** 10.1371/journal.pone.0061236

**Published:** 2013-04-19

**Authors:** Guillermo López, Joaquín Muñoz, Ramón Soriguer, Jordi Figuerola

**Affiliations:** 1 Department of Biodiversity Conservation and Applied Ecology, Estación Biológica de Doñana, Seville, Andalucía, Spain; 2 Department of Wetland Ecology, Estación Biológica de Doñana. Seville, Andalucía, Spain; Universidade Federal de Minas Gerais, Brazil

## Abstract

Earlier migration in males than in females is the commonest pattern in migrating passerines and is positively related to size dimorphism and dichromatism. The early arrival of males is a costly trait that may confer reproductive advantages in terms of better territories and/or mates. Given the physiological cost of migration, early migrants are those in best condition and accordingly the prevalence, load, and/or diversity of parasites is expected to increase in both sexes for late migrants. To test this hypothesis, we sampled 187 trans-Saharan migrant garden warblers *Sylvia borin* and 64 resident serins *Serinus serinus* (as a control for potential circannual patterns in parasite load) during spring migration in Spain. We assessed the prevalence of blood parasites (*Haemoproteus*, *Plasmodium*, and *Leucocytozoon*) and the prevalence and load of intestinal parasites (mainly coccidians and spirurids). The relationship between parasite (prevalence, load, and richness) and the timing of passage through a stopover area was tested using generalized linear models. Protandry occurs in the monomorphic garden warbler and males migrated on average 5.5 days before females. Intestinal parasite richness increased with the date of migration. The timing of migration was unrelated to the presence or load of the other parasite groups analyzed. Our results support the idea that the timing of migration is a condition-dependent trait and suggests that multiple intestinal parasite infestations could delay migration in birds. Even in monomorphic species parasites may play a role in sexual selection by delaying the arrival of the most infected individuals at breeding grounds, thereby further increasing the benefits of mating with early-arriving individuals.

## Introduction

Many bird species fly long distances twice a year in order to procure an optimum availability of resources [1]. Migrations are costly for birds both in terms of energy and mortality and so good body condition is necessary if they are to be successful [2], [3], [4]. An inter-sexual asynchrony in the timing of spring migration is frequent and protandry (i.e. the earlier migration of males than of females) is the commonest such occurrence [5], [6], [7]. Protandry is an innate behavioral trait [8] and is thought to be the result of the different evolutionary pressures operating on males and females [9], [10]. Three main hypotheses have been proposed to explain protandry: (1) the ‘rank-advantage hypothesis’ (male-male competition for the best breeding sites) [11], (2) the ‘mate-opportunity hypothesis’ (male-male competition for maximum mate acquisition) [12], [13], and (3) the ‘susceptibility hypothesis’ (inter-sexual differences in tolerance to adverse conditions) [14]. Consistent with the latter two hypotheses, protandry seems to be positively correlated to both sexual size dimorphism and sexual dichromatism [15], [16]. In other words, males arrive earlier than females in species with more intense sexual selection. Hence, mate competition is supposed to maximize protandry in species with polygynous mating strategies [6], [17]. According to the mate-opportunity hypothesis, the early arrival on the breeding grounds seems to be related to increased reproductive success for both males [13], [18], [19] and females [12], [20], [21]. However, early arrival is costly in terms of survival both during the route and in the breeding grounds, mainly due to the risk of starvation in the event of bad weather and few available resources at the beginning of spring [22], [23], [24]. Consequently it is to be expected that only birds in prime condition opt for this migration tactic. On this basis, conditions on wintering grounds have been shown to affect the timing of arrival in the breeding areas [25], [26], [27].

Parasites may negatively affect host body condition [28], [29], [30]; but see [31], [32], mate attractiveness and plumage brightness [33], [34], [35], reproductive success [36], [37], and survival [28], [38], [39]. However, the impact of parasites on hosts may be strongly context- and species-dependent since not all parasites have the same effect [40] and parasite impacts on host condition may in fact depend on access to resources [41], [42]. Given that migration success is condition-dependent, early arriving individuals would be expected to have lower parasite loads than late arriving individuals. The few studies that have explored the relationship between parasites and the timing of migration in birds are based on a reduced number of parasite groups. For instance, male pied flycatchers *Ficedula hypoleuca* infected with haematozoan *Trypanosoma* were found to arrive on average two days later than non-infected birds [43]. Likewise, barn swallows *Hirundo rustica* infected with *Hirundoecus malleus* (Mallophaga) and *Haemoproteus prognei* (Haematozoa) arrived later in their breeding grounds [14]. However, we are not aware of any published study that had monitored potential phenological-related changes in parasite abundance. The goal of our study was to examine the relationship between the composition and abundance of the community of endoparasites (avian blood and intestinal parasites) and the timing of bird migration. We studied garden warblers (*Sylvia borin*) on spring migration at a stopover area. To control for possible temporal changes in parasite load or richness during the study period, we used the sedentary serin (*Serinus serinus*). Parasites hosted by garden warblers may have infected them at the breeding grounds, at the wintering grounds or at the stopovers [44], [45]. Garden warblers provide an ideal study case given that (1) all birds are strictly migrant in our study area (i.e. they do not breed or winter in the region) and (2) they are abundant during the migration period [46]. Due to the current lack of information, we also tested the assumption that protandry is reduced or does not occur in monomorphic and monochromatic species [47]. Our hypothesis is that parasites negatively affect the timing of migration due to their impact on host body condition. If migration is a condition-dependent process, we would expect to find an increase in parasite prevalence, load, and/or richness during the migratory period. Our results support the hypothesis that at least some parasites may negatively affect timing of migration.

## Materials and Methods

The garden warbler is a passerine that winters in Central and Southern Africa and breeds throughout the Western Palaearctic [46]. Like all trans-Saharan migratory birds, garden warblers migrate long distances (around 6,000 km) and cross vast areas of unsuitable habitats such as sea and deserts [1], performing huge non-stop movements during pre-breeding migration [48]. Thus, their arrival date will depend on the timing of departure, the duration of the stopovers and on the distance between wintering and breeding areas [49]. In order to control for phenological changes in parasite loads that might bias our analyses, we also analyzed a resident species, the European serin, which inhabits similar habitats to the warblers in our study area. The logic behind including data for a resident species is that any relationship between parasites and date in a resident species will indicate that parasitic indexes vary with date independently of migratory phenology.

### Sampling

A total of 187 garden warblers and 64 serins were captured during the 2004 pre-breeding migration period (from March to May) in a tree nursery in the outskirts of Seville, southern Spain (37° 23′ N, 5° 57″W). Birds were trapped from sunrise to sunset using twenty mist nets and were individually marked with numbered aluminum rings. Serins were sexed according to plumage characteristics (Svensson 1994) and garden warblers by molecular markers (see below). Blood (0.1 ml) from 183 garden warblers was taken from the jugular vein using 29 G sterile insulin syringes, placed in vials and stored at –20°C. Afterwards, birds were kept individually in clean cloth bags for 20 minutes to collect fecal samples (0.5–1.0 mg), which were placed in individually marked vials containing 5% formaldehyde. Fecal samples were obtained from 100 garden warblers and 64 serins. All birds were safely released immediately after sampling.

Ringing procedures were approved by the Spanish Ministry of Environment according to Ley 8/2003 (permit number 530394). Blood samples were taken with authorization of the Spanish Ministry of Environment (permit number 39/2003). All efforts were made to minimize suffering during handling and sample collecting, and birds were released in less than 30 minutes after their capture. According to Spanish law in 2004, no approval by Animal Care and Use Committee was needed for this field study (Ley 8/2003).

### Laboratory Methods

Because garden warblers cannot be sexed by plumage characteristics [47], birds were sexed using molecular protocols [50]. DNA extraction followed a standard chloroform/isoamylalcohol method [51]. As well, molecular methods were used to detect and characterize the strains of avian malaria protozoa (i.e. *Haemoproteus*, *Plasmodium*, and *Leucocytozoon*). PCR amplifications were conducted by a nested protocol following Hellgren et al. [52]. Blank DNA extraction and positive and negative controls for PCR were included to detect contamination and false positives/negatives. All positive samples were sequenced using both Haem-F/Haem-R2 and Haem-FL/Haem-R2L primer pairs for *Plasmodium*/*Haemoproteus* and *Leucocytozoon*, respectively. PCR fragments were labeled by the BigDye 1.1 technology (Applied Biosystems). Sequences were resolved using an ABI 3130×l automated sequencer (Applied Biosystems).

Faeces were filtered through a double thickness of cotton-lint cheesecloth and scanned for eggs of endoparasites in a McMaster chamber. For each sample, 200 µl were dried and the dry weight of the feces was used to estimate the number of oocysts or eggs per mg of dry feces [53].

### Statistical Analysis

The timing of migration was expressed as days from March 31 onwards and ranged between 1 and 49. The prevalence (proportion of positive cases in a group of birds) was calculated for all type of parasites, but quantification was only obtained for intestinal parasites. Parasite richness (total number of parasite groups infecting an individual) was estimated separately for blood (i.e. *Plasmodium*, *Haemoproteus*, and *Leucocytozoon*) and intestinal parasites (i.e. coccidians, spirurids, and others). Given that estimates of blood and intestinal parasitic indexes were not available for all individuals, the models for both groups of parasites were run separately. Blood parasite richness and intestinal parasite richness were not correlated (r = −0.01, F_1, 69_ = 0.001, p = 0.999). To test the relationship between the timing of migration and blood parasite prevalence and richness, we fitted a univariate generalized linear model (GLM) using SPSS 17.0 package (SPSS. Inc., Chicago) considering timing of migration as the dependent variable and sex, *Plasmodium*, *Haemoproteus, Leucocytozoon* prevalence (expressed as positive/negative) as factors, and blood parasite richness as covariate. All the two-way interactions between parasite parameters and sex were included in the initial model (see tables 1 and 2 ) and a stepwise backward model selection procedure was followed until all the independent variables remaining in the model increased model fit significantly at *p*-value <0.05. To test the relationship between timing of migration and intestinal parasite load and richness, we performed a GLM including timing of migration as dependent variable, log-transformed intestinal parasite loads (coccidian, spirurids, and other intestinal parasite loads) and intestinal parasite richness as covariates, and sex, and morning/afternoon effect (to make estimates of coccidian load reliable) [53] as factors. Bird gender was forced to remain in the final model. Interactions between morning/afternoon and coccidian load, as well as between sex and the intestinal parasite loads and richness were included in the initial model. Finally, a similar analysis was conducted with the serin dataset. Richness was not included in the serin analysis since only coccidians were found in serins.

**Table 1:** Relationships between migration time and blood parasite prevalence and richness.

Source	Df	F	p
**Sex**	**1, 181**	**15.41**	**<0.001**
*Leucocytozoon* prevalence	1, 180	1.18	0.28
Blood parasite richness	1, 180	037	0.54
*Haemoproteus* prevalence	1, 180	0.04	0.85
*Plasmodium* prevalence	1, 180	1.27	0.26
*Leucocytozoon* prevalence*Sex	2, 179	1.80	0.17
Blood parasite richness*Sex	2, 179	0.19	0.83
*Plasmodium* prevalence*Sex	2, 179	1.13	0.33
*Haemoproteus* prevalence*Sex	2,179	1.97	0.14

Results of the general linear models testing the relationships between ringing dates (as dependent variable) and blood parasite prevalence and richness. Sex was included as an independent factor. Data from variables in the final model are presented in bold. For variables not in the final model the significance when added to the model is given.

**Table 2:** Relationships between migration time and blood parasite prevalence and richness.

Source	Df	F	p
**Sex**	**1, 97**	**1.97**	**0.16**
**Intestinal parasite richness**	**1, 97**	**8.65**	**0.004**
Log (Spirurid load)	1, 96	2.33	0.13
Morning/afternoon	1, 96	0.58	0.54
Log (Coccidian load)	1, 95	0.38	0.54
Log (“Others” load)	1, 96	<0.01	0.98
Sex*Intestinal parasite richness	1, 96	2.26	0.14
Morning/afternoon* Coccidian load	2, 95	0.05	0.95
Sex*“Others” load	2, 95	0.29	0.75
Sex*Coccidian load	2, 94	0.29	0.75
Sex*Spirurid load	2, 95	1.29	0.28

Results of the general linear models testing the relationships between ringing dates (as dependent variable) and intestinal parasite load and richness. Sex was included as an independent factor. Data from variables in the final model are presented in bold. For variables not in the final model the significance when added to the model is given.

## Results

Blood parasites were detected in 66.1% of the 183 garden warblers sampled. The prevalence of *Plasmodium*, *Haemoproteus*, and *Leucocytozoon* were 15.8% (six different strains), 45.4% (six different strains), and 14.2% (five different strains), respectively (see Table 3). A total of 58.5% of the birds (87.7% of the infected individuals) were infected with just a single blood parasite species. Moreover, two *Plasmodium*-positive specimens were infected by more than one strain. Finally, 14 specimens were co-infected by more than one parasite genus (Table 3). Prevalence and richness of blood parasites were unrelated to the timing of migration (Table 1).

**Table 3:** Blood parasite genus and strains.

Plasmodium				
Genetic lineage	GB Acc. Num.	Reference	#	Comment
Delurb5	EU154347	Marzal et al. J. Evol. Biol. (2008)	1	
KS-2006-1	DQ356303	GenBank	2	
*P. nucleophilum*	AF254962	Bensch et al. Proc. R. Soc. Lond., B, Biol. Sci.(2000);Perkins & Schall. J. Parasitol. (2002)	1	
Rinshi-1; SGS1 (*)	AB458849; AF495571	Kim et al. Parasitol. Res. (2009);Waldenstrom et al. Mol. Ecol. (2002)	15	
Rinshi-11	AB477124	Kim et al. Parasitol. Res. (2009)	2	
RTSR1	AF495568	Waldenstrom et al. Mol. Ecol. (2002)	1	Based on 474 bp fragment
Mixture unidentifiable lineages			2	
Haemoproteus				
COLL2	FJ355915	GenBank	1	
SYBOR1	AF495575	Waldenstrom et al. Mol. Ecol. (2002)	62	
SYBOR1.SPAIN	KC682871	This study	1	Differs with SYBOR1 in one position based on 497 bp fragment
*SYBOR15*	EF032812	GenBank	2	
SYBOR3	DQ368365	Perez-Tris et al. PLoS ONE (2007)	1	
WW1	AF254971; AY099038	Bensch et al. Proc. R. Soc. Lond., B, Biol. Sci.(2000);Perkins & Schall. J. Parasitol. (2002)	1	
Plasmodium & Haemoproteus				
Mixture Rinshi-1; SGS1 & SYBOR1			3	
Leucocytozoon				
NEHUM01.SPAIN	KC682872	This study	2	Differs with NEHUM01 (JN032625)in four positions
SFC8	DQ847234	GenBank	1	
SYBOR14; SYBOR6	DQ847241; DQ847237	GenBank	3	
SYBOR14; SYBOR6.SPAIN	KC682873	This study	1	Differs with SYBOR14; SYBOR6 inone position
SYBOR7; SYBOR13	DQ847238; DQ847232	GenBank	6	
SYBOR7; SYBOR13 & SYBOR14; SYBOR6			1	
Haemoproteus & Leucocytozoon				
SYBOR1.SPAIN & SYBOR14; SYBOR6.SPAIN			1	
Padom5, SYBOR1 & SYBOR14;SYBOR6	HM146898 (Padom5)	GenBank	1	
SYBOR1, unidentifiable Haemoproteuslineage & SYBOR7; SYBOR13			1	
SYBOR1 & SYBOR14; SYBOR6			5	
SYBOR1 & NEHUM01.SPAIN			1	
Plasmodium & Leucocytozoon				
Rinshi-1; SGS1 & SYBOR14; SYBOR6			1	
Rinshi-1; SGS1 & SYBOR7; SYBOR13			1	
Haemoproteus, Plasmodium& Leucocytozoon				
SYBOR1, KS-2006-1, SYBOR14; SYBOR6& SYBOR7; SYBOR13			1	
		Total #of infected individuals	121	

Different blood parasite genus and strains found in this study with their data deposition in GenBank (GB), accession numbers, references and additional comments. Note that strains are identified for *Plasmodium* and *Haemoproteus* genus with a Cyt-b lenght fragment of 478 bp, and for *Leucocytozoon* genus with a length fragment of 467 bp (but for NEHUM01 with 476 bp length). Lineages labeled with (*) have the same nucleotide sequence in GB, but their descriptive names and accession numbers are different.

**Figure 1:**
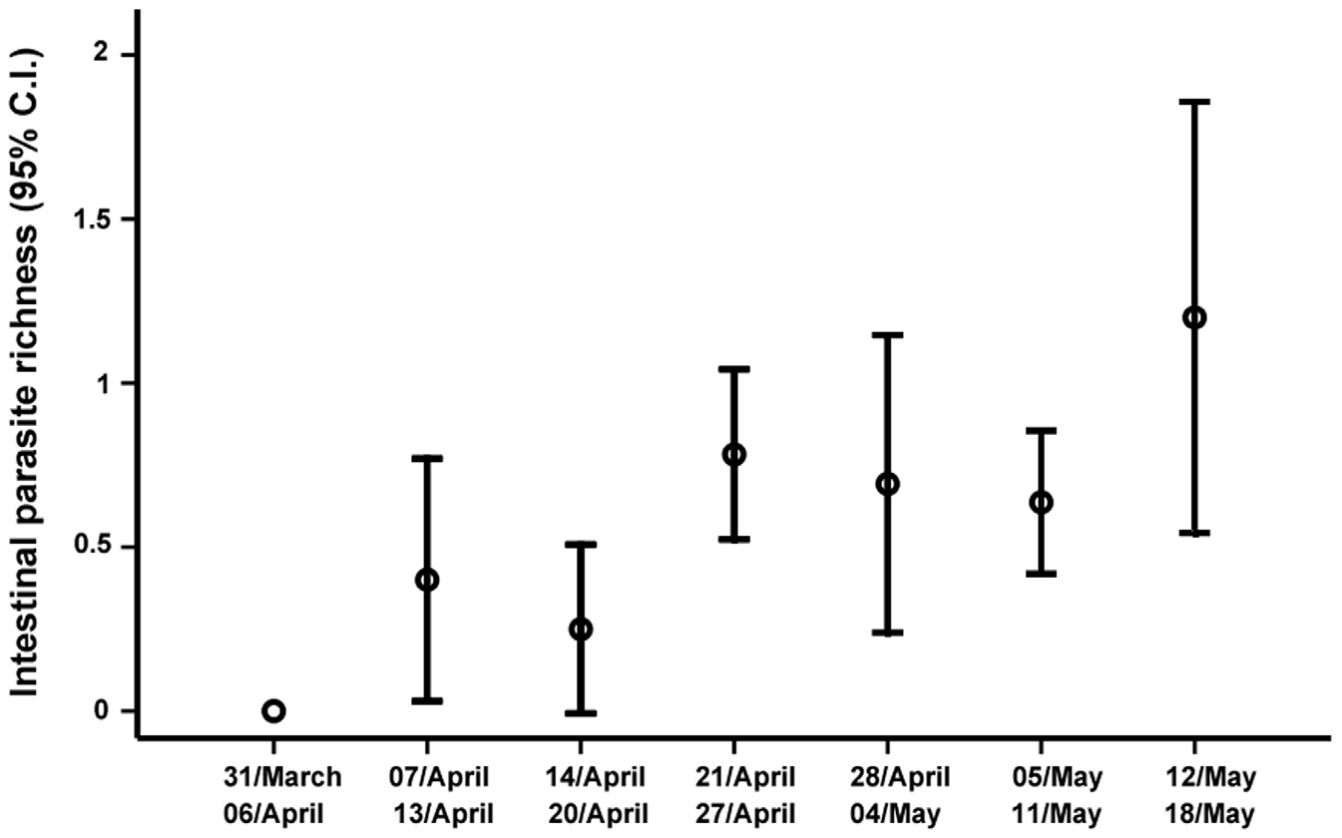
Changes in intestinal parasite richness. Mean and 95% confidence interval for intestinal parasite richness in the seven weeks of the study period.

Intestinal parasites were found in 68% of the 100 fecal samples of garden warbler analyzed. The most frequent parasite species found were Protozoan coccidia identified as *Isospora* spp. and Nematoda spirurids identified as *Tetrameres*-like. *Isospora* are monoxenous intestinal protozoan [54], whereas *Tetrameres* are heteroxenous proventricular nematodes [55]. Some other species of trematodes, cestodes, and nematodes were included in the analyses grouped as ‘other parasites’ since they were found with a prevalence of less than 0.1%. The prevalence of *Isospora*, spirurids, and ‘other parasites’ reached 35%, 13.6%, and 13.6%, respectively. In total, 43.2% of garden warblers were infected with a single intestinal parasite group, 22.5% with two, and 3.9% with all three groups. In serins, only the coccidian *Isospora* spp. was found with a prevalence of 39.1%. Intestinal parasite richness was positively related to the timing of migration in garden warblers (Table 2; Fig. 1). The coccidian load was unrelated to date in serins (F_1, 59_ = 0.05; p = 0.82).

**Figure 2:**
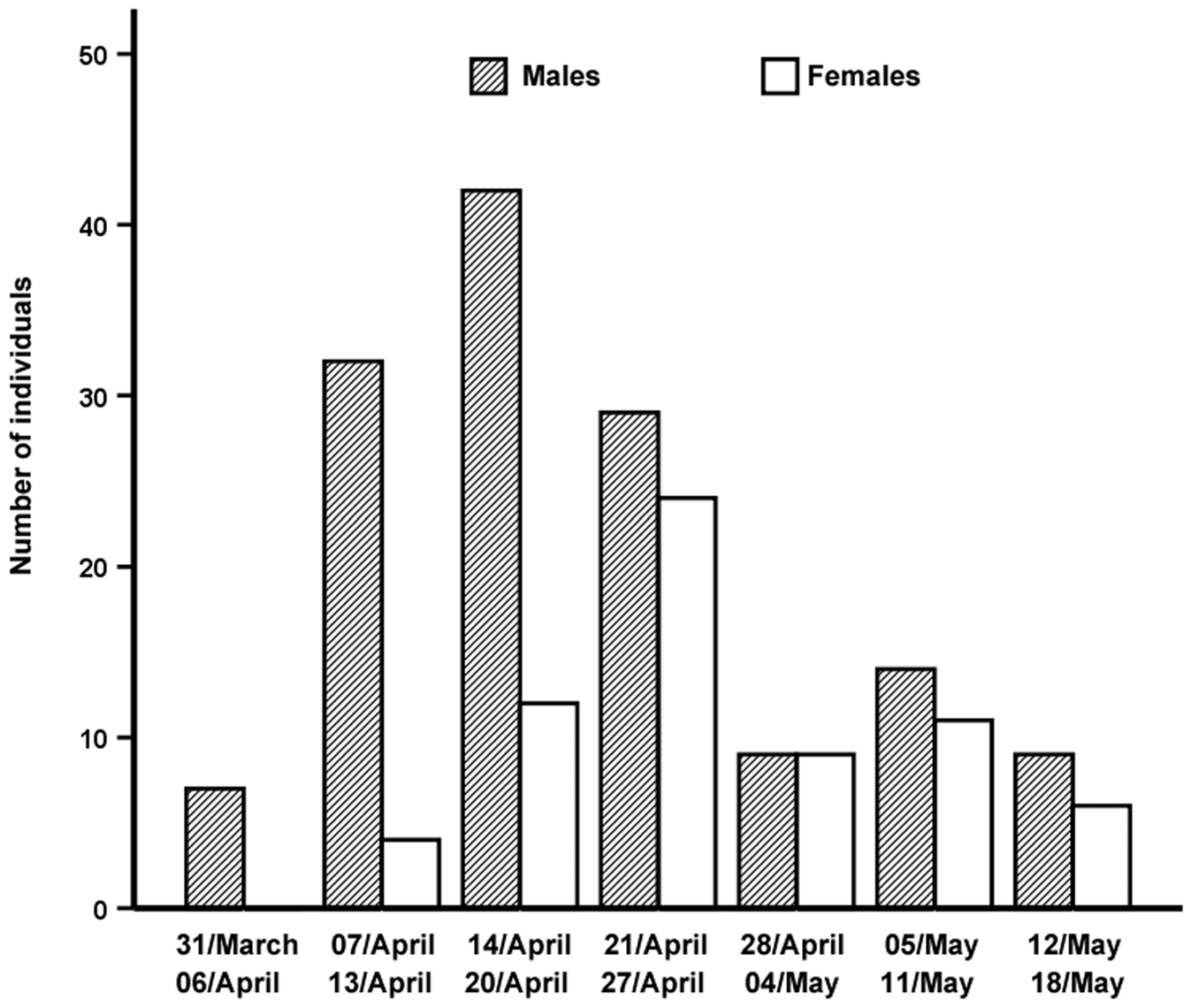
Sexual differences in timing of migration. Number of males (barred bars) and females (empty bars) Garden Warblers captured in each of the seven weeks of the study period.

Finally, males arrived significantly earlier than females independently of their blood parasite prevalence or richness (arrival mean day ± standard error: 24.28±1.45 *vs.* 29.74±1.92; n = 183, Fig. 2).

## Discussion

To the best of our knowledge, protandry had not been reported in a species lacking sexual size dimorphism or dichromatism. Our results show that protandry occurs in the garden warbler and males migrate on average 5.5 days ahead of females. This result highlights the need to incorporate data from monomorphic species on the comparative studies of the causes and consequences of protandric behavior. Interestingly, the difference in mean time of passage between males and females is one of the largest reported both for a trans-Saharan passerine and for a warbler from the genus *Sylvia* [16].

We failed to detect any significant relationship between blood parasites and the timing of migration. This suggests that blood parasites only have a minor impact on arrival dates in our study system, at least in comparison with the effects of intentinal parasites (note that sample size for both groups of parasites are high [56]). Our findings contrast to Rätti et al. [43], likely due to (1) the different protozoan genera found in each study (*Trypanosoma* is considered to produce stronger negative effects on host health than either *Plasmodium*, *Haemoproteus*, or *Leucocytozoon* [57], [58]) or to (2) a non-linear effect of blood parasites, with effects that may show up at or near the final destination and be negligible in a stopover area. Our results support the hypothesis that migration phenology is a condition-dependent trait and that there is a significant relationship between intestinal parasite richness and arrival dates (see Table 2). It is unlikely that the differences in passage time between males and females were related to differences in parasite richness, because the interaction between both variables was not significant. Under this scenario, the temporal gradient found in the richness of intestinal parasites could be caused by (1) earlier migration by individuals infected with fewer intestinal parasite species, (2) faster migration by less parasitized individuals, (3) a decrease in the ability to control intestinal parasites in individuals in the poorest condition that migrate later than those in prime condition, and/or (4) a north-south gradient in intestinal parasite load and diversity in wintering grounds (individuals wintering in the north of the wintering grounds being less parasitized and arriving earlier to the stopover area than those wintering in the south). Given both the broad distribution of intestinal parasites [54] and the diversity of wintering grounds [56], the last hypothesis seems unlikely. Thus, the positive correlation between intestinal parasite richness and the timing of migration could be caused by a combined negative effect of different intestinal parasite species on individual health status. Coccidians affect resource availability in hosts and thus hinder nutrient absorption [59], [60], [61], having little effect when resources are abundant [42], [62] but negative effects when resources are scarce [63], [64]. In fact, coccidian load was also positively related to passage date in the garden warblers in univariate analyses (F_1, 96_ = 4.67, p = 0.03), but this effect disappeared after controlling for parasite richness (see results). The negative results obtained with the resident serins in the same period exclude a confounding effect produced by a potential change in parasite richness or coccidian load through the spring. Given that the breeding cycle of serins is different from that of the garden warbler, our sampling period could not provide sufficient comparison base. It is important to note that serins were already breeding during the study period, and this may have obscured potential comparisons. However, all resident species were breeding at that time, and no better alternatives exist. Spirurids of the genus *Tetrameres* provoke gastric dysfunction when parasitic loads are high [65], [66]. The group catalogued as ‘other parasites’ includes many different intestinal parasites with potential effects on, for instance, digestive and respiratory dysfunctions [40]. Our results seem to support the theory that birds display lower ability to control multiple infestations than single ones. Increases in energy demands make individuals less able to control their infections [67]. Thus, higher intestinal parasite richness in late migrants may also be a consequence of the poorer condition of these migrants since they have to devote energies to limiting the effects of these pathogens [55], [68].

Overall, our results show that (1) protandry may occur in monomorphic passerines, (2) that timing of arrival at a stopover area seems influenced by parasite load, and that (3) by mating with early arriving males females will not only obtain the best territories [13] and/or parents [11] but also the mates with fewer parasites. Such results suggest that parasites may have an indirect effect on mate choice, even in species that are not brightly colored or have no obvious ornaments. Consequently, further observational and experimental studies on the likely influence of intestinal parasite richness on the fitness consequences of individual migration behavior are necessary if we are to shed light on the evolution of migration patterns in long-distance migration behavior in passerine birds.
